# Significance of Coronary Artery Spasm Diagnosis in Patients With Early Repolarization Syndrome

**DOI:** 10.1161/JAHA.117.007942

**Published:** 2018-02-07

**Authors:** Tsukasa Kamakura, Mitsuru Wada, Kohei Ishibashi, Yuko Y. Inoue, Koji Miyamoto, Hideo Okamura, Satoshi Nagase, Takashi Noda, Takeshi Aiba, Satoshi Yasuda, Wataru Shimizu, Shiro Kamakura, Kengo Kusano

**Affiliations:** ^1^ Division of Arrhythmia and Electrophysiology Department of Cardiovascular Medicine National Cerebral and Cardiovascular Center Suita Osaka Japan; ^2^ Department of Cardiovascular Medicine Nippon Medical School Tokyo Japan

**Keywords:** coronary spasm, early repolarization syndrome, ventricular fibrillation, Sudden Cardiac Death, Ventricular Fibrillation

## Abstract

**Background:**

Previously described patients with early repolarization syndrome (ERS) may have experienced silent coronary artery spasm (CAS) because the diagnosis of CAS was mainly based on symptoms or coronary angiography findings, without performing a spasm provocation test. This study investigated the significance of CAS diagnosis and evaluated the incidence of silent CAS in patients with possible ERS (ie, idiopathic ventricular fibrillation [VF] and inferolateral J wave).

**Methods and Results:**

The study included 34 patients with idiopathic VF and inferolateral J wave. Thirteen patients (38%) were diagnosed as having CAS on the basis of coronary angiography with spasm provocation test (n=8) and documentation of spontaneous ST elevation (n=5). Of the 13 patients with CAS, 5 (38%) did not experience chest symptoms before and during VF, and were diagnosed as having silent CAS. The remaining 21 patients (62%), with a negative provocation test result and absence of chest symptoms, were considered to have ERS. During the 92 months of follow‐up, patients with CAS receiving appropriate medical treatment with antianginal drugs showed a favorable outcome. In contrast, 4 of 21 patients with ERS (19%) had VF recurrences. The use of monotherapy or combination therapy, consisting of quinidine, cilostazol, and bepridil, in the 4 patients with ERS, was effective in suppressing VF.

**Conclusions:**

Approximately 40% of patients with CAS with documented VF and inferolateral J wave did not experience chest symptoms at the first VF, and could have been misdiagnosed as having ERS. The use of the spasm provocation test is considered essential to differentiate patients for optimal medical treatment.

**Clinical Perspective**

**What Is New?**

- Both early repolarization syndrome (ERS) and coronary artery spasm (CAS) may induce ventricular fibrillation in patients without structural heart disease.
- Differentiating CAS from ERS as a cause of ventricular fibrillation may be challenging because patients with CAS are often associated with inferolateral J wave, mimicking ERS.
- This was the first study evaluating the incidence of CAS, in particular silent CAS, in patients with possible ERS.

**What Are the Clinical Implications?**

- The results of this study suggest that a considerable number of patients previously diagnosed as having ERS may have been cases of silent CAS unless a spasm provocation test was performed.
- Treatment with antianginal drugs is essential to prevent the recurrence of ventricular fibrillation in patients with CAS, whereas use of quinidine, cilostazol, and bepridil was effective in patients with pure ERS.
- The spasm provocation test and close clinical follow‐up are considered essential to distinguish patients with silent CAS from those with ERS.

Early repolarization syndrome (ERS) is defined as inferolateral J wave after resuscitation from idiopathic ventricular fibrillation (VF), in the absence of other causes of cardiac arrest, such as coronary artery disease.^1^, ^2^

Coronary artery spasm (CAS) is a functional impairment that may cause VF in patients without structural heart disease. The prevalence of CAS in patients resuscitated from cardiopulmonary arrest ranges from 3.3% to 8.6%.^3^, ^4^, ^5^, ^6^ It is known that CAS may induce fatal ventricular arrhythmias without apparent chest symptoms.^7^, ^8^ In addition, inferolateral J wave has been associated with an increased risk of fatal ventricular arrhythmias in patients with CAS.^9^, ^10^, ^11^ This indicates that silent CAS (ie, CAS without any symptoms before VF) may have been present in previously reported cases of ERS.

In previous studies of ERS, the diagnosis of CAS was limited, determined on the basis of symptoms and findings of coronary angiography (CAG), without performing a spasm provocation test. In a multicenter study involving patients with ERS, a spasm provocation test was performed in only 19 of 122 patients (16%).^12^ The clinical management of CAS and ERS is different; therefore, it is important to differentiate the 2 conditions. However, the incidence of CAS (in particular, silent CAS) among patients with possible ERS (ie, idiopathic VF and inferolateral J wave) remains unknown.

The objective of this study was to investigate the significance of CAS diagnosis and evaluate the incidence of silent CAS in patients with possible ERS.

## Methods

The data, analytic methods, and study materials will not be made available to other researchers for purposes of reproducing the results or replicating the procedure.

### Study Population

The study included 34 patients with idiopathic VF and inferolateral J wave, admitted to the National Cerebral and Cardiovascular Center (Osaka, Japan) between 1996 and 2016. None of the patients had structural heart disease, which was ruled out by noninvasive investigation (physical examination, 12‐lead electrocardiography, Holter electrocardiography, exercise stress test, signal‐averaged electrocardiography, and cardiac magnetic resonance imaging or computed tomography) and invasive angiography, including CAG and right or left ventricular cineangiography. Patients with Brugada syndrome, long/short‐QT syndrome, catecholaminergic polymorphic ventricular tachycardia, commotio cordis, drug‐induced VF, or hypothermia were excluded. Patients requiring catheter ablation because of frequent premature ventricular contractions (≥1000/day) originating from the Purkinje network or the ventricular outflow tract were also excluded. An abnormal corrected QT interval was defined as <340 and ≥460 ms during sinus rhythm. This study was approved by the Institutional Research Board of the National Cerebral and Cardiovascular Center. All participants gave written informed consent.

### Classification of Each Group

**Figure 1:**
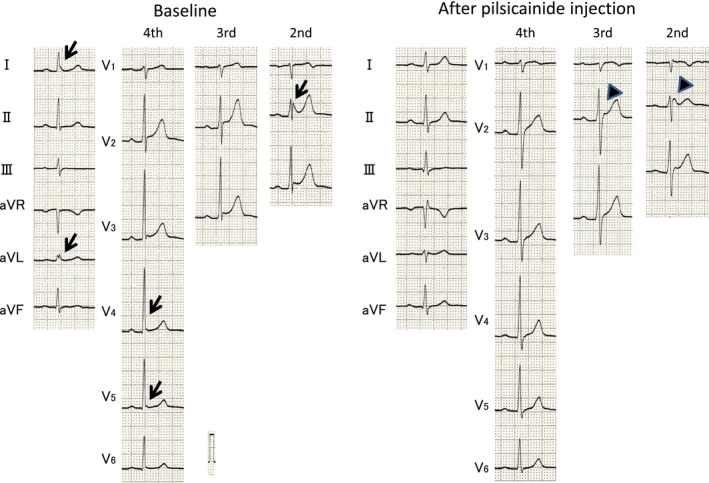
At baseline, ECGs of a 42‐year‐old man in the early repolarization syndrome group exhibited J waves (arrows) followed by ascending ST segments in leads I, aVL, V_4_, and V_5_ in the standard (fourth) recording and in lead V_2_ in the second intercostal recording. After 50‐mg pilsicainide injection, all J waves in limb lead disappeared. In addition, saddleback ST elevation with slightly augmented J waves (broad arrows) were noted in V_2_ in the high (second and third) intercostal spaces.

**Figure 2:**
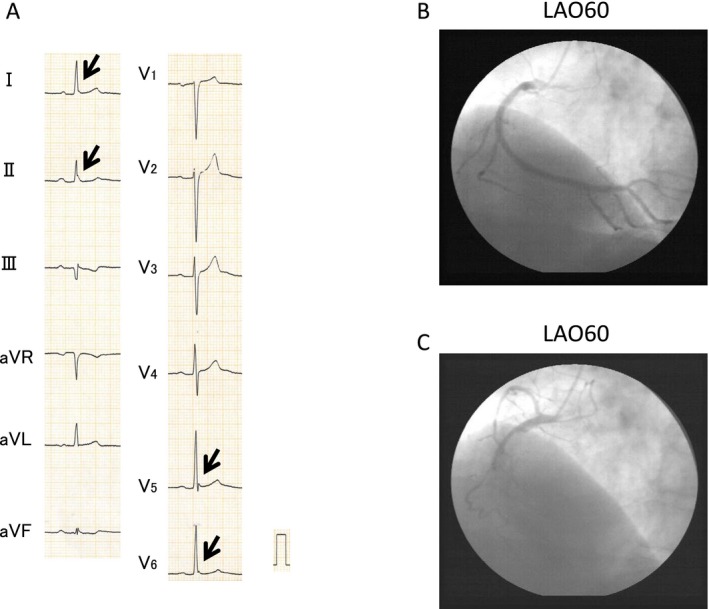
A, ECGs of a 54‐year‐old man exhibited J waves in leads I, II, V_5_, and V_6_ (arrows). The patient experienced ventricular fibrillation during sleep in the absence of chest symptoms. B, No significant coronary stenosis was found in control coronary angiography before spasm provocation tests. C, Coronary artery spasm (CAS) was induced in the right coronary artery by intracoronary artery injection of ergonovine accompanied by chest pain. The patient was diagnosed as having silent CAS. LAO indicates left anterior oblique.

**Figure 3:**
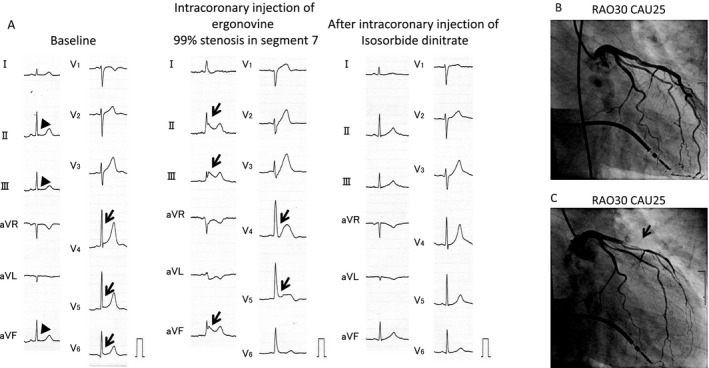
A, At baseline (left), ECGs of a 49‐year‐old man exhibited J waves ≧1 mm in leads V_4_, V_5_, and V_6_ (arrows) and J waves <1 mm in leads II, III, and aVF (broad arrows). ECGs during the spasm provocation test (middle) showed marked J‐wave augmentation in leads II, III, and aVF and ST elevation accompanied by QRS widening in leads V_4_ and V_5_ (arrows). These resolved after intracoronary infusion of isosorbide dinitrate (right). B, There were no significant stenotic lesions observed in both coronary arteries. C, After intracoronary injection of ergonovine, 99% stenosis was provoked in the left ascending artery (segment 7). During provocation of severe stenosis, the patient reported chest pain. CAU indicates caudal; and RAO, right anterior oblique.

**Figure 4:**
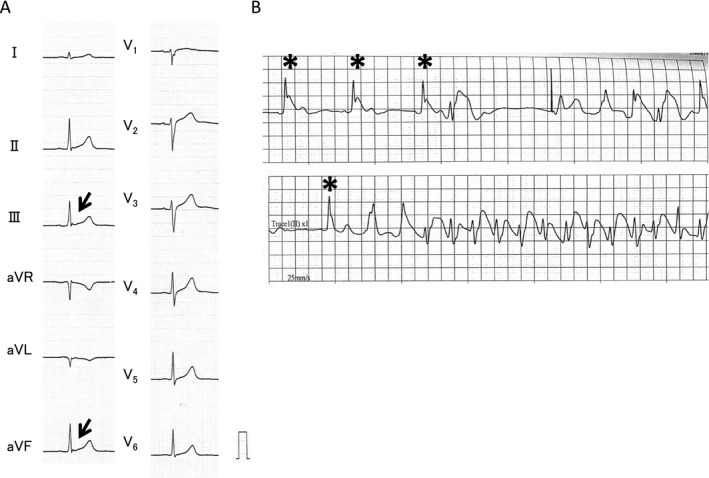
A, ECGs from a 59‐year‐old man in the coronary artery spasm group showed notched J waves in leads III (1.1 mm) and aVF (1.0 mm) (arrows). The patient felt slight chest discomfort before the first ventricular fibrillation (VF). B, After admission to the hospital, nonsustained ventricular tachyarrhythmia associated with chest pain was observed in the monitoring ECG. Immediately before the onset of nonsustained ventricular tachyarrhythmia, ECGs showed dynamic J‐wave activity (5.8 mm) accompanied by notching and QRS widening (asterisks), which are consistent with typical electrocardiographic features before VF observed in previous studies of patients with early repolarization syndrome.

Thirty‐four patients with idiopathic VF and inferolateral J wave were divided into 2 groups according to the presence of CAS: (1) the ERS group (Figure 1) and (2) the CAS group (Figures 2, 3, 4 through 5). The diagnosis of CAS was based on spontaneous angina attacks or a total or subtotal coronary artery narrowing (>90%) during the CAG, accompanied by ischemic electrocardiographic changes and/or chest pain, either spontaneously or in response to a provocative stimulus (eg, acetylcholine, ergonovine, or hyperventilation).^13^ The spasm provocation test was performed according to the method included in the Guidelines for Diagnosis and Treatment of Patients with Vasospastic Angina of the Japanese Circulation Society.^13^ Briefly, after confirming the absence of significant stenotic lesions in both right and left coronary arteries, incremental doses of acetylcholine or ergonovine were injected into the left coronary artery (acetylcholine, 20, 50, and 100 μg; ergonovine, 20, 40, and 60 μg) and the right coronary artery (acetylcholine, 20 and 50 μg; ergonovine, 20, 40, and 60 μg) until coronary vasospasm was detected angiographically or until the maximum dose was administered. The definition of spontaneous attack was an angina at rest and/or effort, accompanied by a transient ST‐segment elevation or depression of >0.1 mV, or a new appearance of negative U wave on electrocardiography. Silent CAS was diagnosed in patients without chest symptoms before VF or spasm provocation test, and in those meeting the diagnostic criteria for CAS after coronary evaluation (Figure 2).

**Figure 5:**
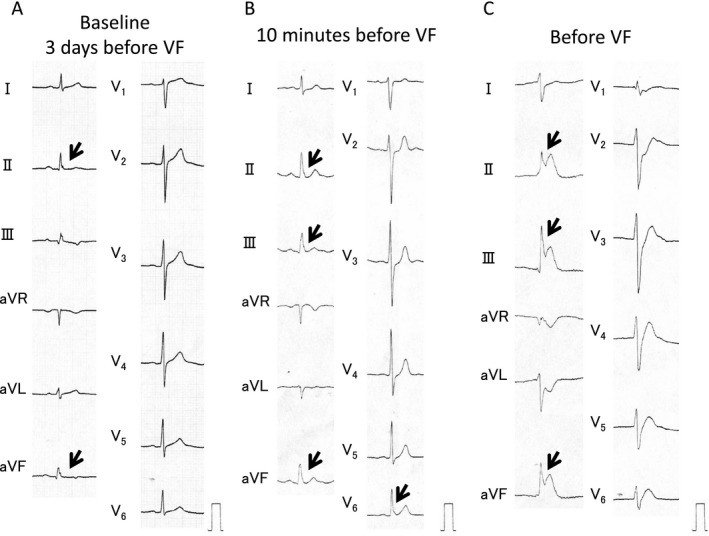
A, At baseline, ECGs of a 44‐year‐old man in the coronary artery spasm group exhibited J waves, followed by horizontal ST segments in leads II (1.4 mm) and aVF (1.7 mm) (arrows). B, ECGs 10 minutes before ventricular fibrillation (VF) showed slight augmentation of J waves and QRS widening in leads II (2.3 mm), III (2.0 mm), aVF (2.3 mm), and V_6_ (1.5 mm) (arrows). The patient complained of chest pain at that time. C, ECGs before VF showed marked J‐ST elevation and QRS widening in leads II (4.7 mm), III (6.3 mm), and aVF (5.7 mm), which are consistent with typical electrocardiographic features before VF observed in previous studies of patients with early repolarization syndrome.

The presence of inferolateral J wave, defined as an elevation of the J point in ≥2 contiguous leads, was evaluated at baseline using 12‐lead electrocardiography (25 mm/s and 10 mm/mV).^1^, ^14^ The amplitude of the inferolateral J wave or J‐point elevation had to be ≥1 mm or 0.1 mV above the baseline level, either as QRS slurring or notching in any of the inferior (II, III, and aVF), lateral (V_4_, V_5_, and V_6_), and high lateral (I and aVL) leads in at least 1 electrocardiographic recording. The QRS interval in patients with inferolateral J wave had to be <120 ms.

An anterior J wave was defined as saddleback‐type electrocardiography (type 2 and type 3 Brugada‐pattern electrocardiography) or upward/downward notching or downward slurring with an amplitude of ≥1 mm at the end of the QRS to early ST segment in any anterior leads (V_1_, V_2_, and V_3_) in the baseline standard or high intercostal (second and third) electrocardiographic recordings or after drug provocation tests.^15^ None of the ECGs of patients with ERS revealed a type 1 Brugada‐pattern ECG, including in high intercostal spaces.

Brugada syndrome was diagnosed by the presence of type 1 ST‐segment elevation, occurring spontaneously or after intravenous administration of a sodium channel blocker, in at least 1 right precordial lead (V_1_ and V_2_), placed in a standard or superior position (up to the second intercostal space).^16^ Type 1 electrocardiography was defined as a coved‐type J point or ST elevation of ≥2 mm, followed by a negative T wave.

All ECGs were recorded at 25 mm/s and 10 mm/mV. After independent analyses of the electrocardiographic recordings by 3 cardiologists (T.K., K.K., and S.K.), a consensus was reached on the diagnosis for each patient.

### Clinical Data

Clinical data were collected from all patients, including age at the first episode of VF, sex, family history of sudden cardiac death <45 years of age, chest symptoms before the first VF, medical history, patient's activity during VF, prognosis, and drug therapy. The patient's state during VF was defined as “sleep” when the VF occurred in a state of sleep or as “near sleep” when the VF occurred in a resting state immediately after waking up. During follow‐up, patients were considered to have an arrhythmic event if VF was documented by interrogation of an implantable cardioverter defibrillator (ICD). An electrical storm was defined as ≥3 VF episodes within 24 hours. The follow‐up commenced at the time of the first VF event. In patients with recurrent arrhythmias, the selection of antianginal and antiarrhythmic drugs was decided by the treating physician.

Patients were followed up every 3 to 6 months for clinical review and device interrogation. Clinical profiles, electrocardiographic characteristics, and VF recurrences during the 92±62 months of follow‐up were compared between the 2 groups.

### Statistical Analysis

Data were analyzed using the JMP10 software (SAS Institute Inc, Cary, NC). Numeric values are presented as means±SD. Statistical significance was determined using the χ^2^ test or the Student *t* test, as appropriate. Survival curves were constructed using the Kaplan‐Meier method and compared using the log‐rank test. *P*<0.05 was considered a statistically significant difference between groups.

## Results

### Clinical Characteristics of the Study Population

**Figure 6:**
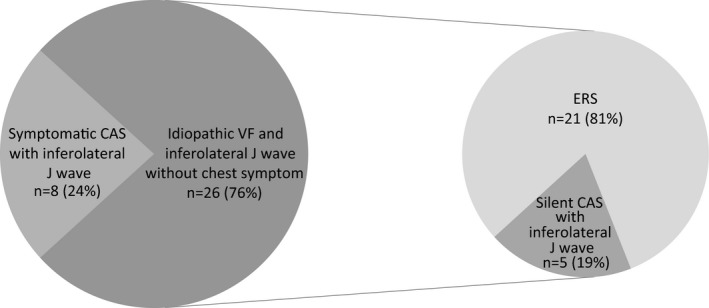
Among 34 patients with idiopathic ventricular fibrillation (VF) and inferolateral J wave, 8 were diagnosed as having symptomatic coronary artery spasm (CAS) by spasm provocation test or spontaneous ST elevation. Of the remaining 26 asymptomatic patients with VF who might have been diagnosed as having early repolarization syndrome (ERS), 5 (19%) had silent CAS. ERS consisted of 9 patients with inferolateral and anterior J‐wave elevations and 12 patients with J‐wave elevations in the inferolateral leads only.

CAG before the spasm provocation test or at convalescent phase after VF demonstrated that none of the 34 patients had significant stenotic lesions. Ergonovine/acetylcholine provocation tests were performed in 29 patients who did not show spontaneous ST elevation at the time of VF or chest symptoms. In the remaining 5 patients, spasm provocation tests were not performed because spontaneous ST elevation accompanied by chest pain was documented at the first VF. Significant coronary vasospasms were induced by acetylcholine (n=6) or ergonovine (n=2) in the left anterior descending (n=5), left circumflex (n=1), and right coronary (n=5) arteries in 5 patients who remained asymptomatic until the provocation test (Figure 2) and 3 patients with chest symptoms before or after VF (Figure 3). Multivessel spasm was documented in 2 patients. Accordingly, 13 of 34 patients (38%) with documented VF and inferolateral J waves were diagnosed as having CAS by spasm provocation tests or spontaneous ST elevation. Of those patients, 5 (38%) were classified as silent CAS cases and the remaining 8 were classified as symptomatic CAS cases. The remaining 21 patients (62%) with negative spasm provocation test results, including 9 patients with J waves in inferolateral and anterior leads and 12 patients with J waves only in inferolateral leads, were considered ERS cases. After all, in 26 asymptomatic patients with VF who might have been diagnosed as having ERS, 5 (19%) were shown to have silent CAS (Figure 6). Comparisons of clinical characteristics between patients in the CAS and ERS groups are shown in the Table. Most patients in both groups were men. Moreover, patients with CAS were significantly older than patients with ERS at the first VF (*P*=0.024).

**Table 1:** Clinical Characteristics of Each Group

Characteristics	Symptomatic CAS (n=8)	Silent CAS (n=5)	CAS (n=13)	ERS (n=21)	*P* Value (Silent CAS vs ERS)
Clinical					
Age at first VF, y	56.8±10.1	49.0±8.4	53.8±9.9^a^	42.7±17.4	0.26
Male sex, n (%)	8 (100)	5 (100)	13 (100)	17 (81)	0.56
FH of SCD, n (%)	0 (0)	0 (0)	0 (0)	3 (14)	1.0
Chest symptoms before VF, n (%)	8 (100)	0 (0)	8 (62)^a^	0 (0)	1.0
Hypertension, n (%)	4 (50)	2 (40)	6 (46)	3 (14)	0.24
Dyslipidemia, n (%)	3 (38)	2 (40)	5 (38)	3 (14)	0.24
Diabetes mellitus, n (%)	1 (13)	2 (40)	3 (23)^a^	0 (0)	0.031
Current smoking, n (%)	7 (88)	3 (60)	10 (77)	10 (48)	1.0
Activity at the time of SCA					
Sleep, near sleep n (%)	1 (13)	1 (20)	2 (15)	8 (38)	0.63
Physical activity, n (%)	3 (38)	2 (40)	5 (38)	6 (29)	0.63
Arousal at rest, n (%)	4 (50)	2 (40)	6 (46)	7 (33)	1.0
Electrocardiographic findings					
Location of J waves					
Inferior (II, III, aVF), n (%)	8 (100)	4 (80)	12 (92)	16 (76)	1.0
Lateral (V_4_–V_6_), n (%)	3 (38)	4 (80)	7 (54)	18 (86)	1.0
High lateral (I, aVL), n (%)	2 (25)	4 (80)	6 (46)	7 (33)	0.13
Anterior (V_1_–V_3_), n (%)	0 (0)	0 (0)	0 (0)^a^	9 (43)	0.13
Extensive (inf+lat/high lat), n (%)	3 (38)	3 (60)	6 (46)	14 (67)	1.0
Amplitude of J point, mm	1.59±0.68	1.30±0.57	1.48±0.63	1.68±0.68	0.24
Clinical outcome					
Follow‐up, mo	60±48	88±62	70±53	105±64	0.59
ICD implantation, n (%)	7 (88)	5 (100)	12 (92)	20 (95)	1.0
VF recurrence, n (%)	3 (38)	0 (0)	3 (23)	4 (19)	0.56
Electrical storm, n (%)	0 (0)	0 (0)	0 (0)	2 (9)	1.0

Data are given as mean±SD unless otherwise indicated. CAS indicates coronary artery spasm; Chest symptoms before VF, chest symptoms include those experienced just before the occurrence of VF and those reported in the patient's medical history; Electrical storm, ≥3 VF episodes within 24 hours; ERS, early repolarization syndrome; FH of SCD, family history of sudden cardiac death before 45 years of age; ICD, implantable cardioverter defibrillator; Inf, inferior lead; Lat, lateral lead; SCA, sudden cardiac arrest; and VF, ventricular fibrillation.

a *P*<0.05 (CAS vs ERS).

### Electrocardiographic Features Before VF in Patients With CAS

ECGs before or at the time of VF were obtained from 3 symptomatic patients with CAS (Figures 4 and 5). In these patients, ECGs were recorded at the time of chest pain, and the patients were subsequently diagnosed as having CAS. In these patients with CAS, specific J‐wave and QRST characteristics, such as J‐point augmentation, QRS widening, and J‐ST elevation, which are considered typical electrocardiographic changes before VF in patients with ERS, were observed just before VF.

### Clinical Outcome

The mean follow‐ups for the CAS and ERS groups were 70±53 and 105±64 months, respectively. Of the 34 patients, 32 (94%) received an ICD. One patient in the CAS group and one patient in the ERS group refused ICD insertion and were followed up without ICD.

**Figure 7:**
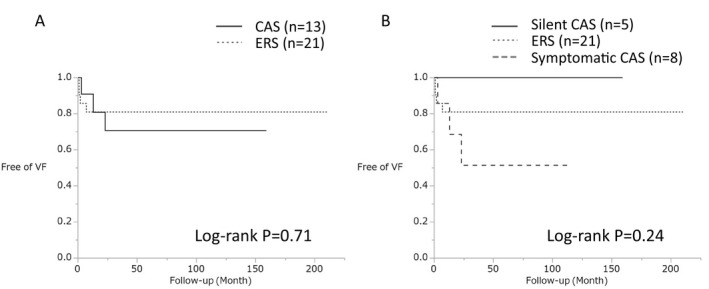
A, Kaplan‐Meier analyses of freedom from ventricular fibrillation (VF) during follow‐up in the early repolarization syndrome (ERS) and coronary artery spasm (CAS) groups. The incidence of VF during follow‐up was similar between the 2 groups. B, Kaplan‐Meier analyses of freedom from VF during follow‐up in the ERS, silent CAS, and symptomatic CAS groups. The incidence of VF during follow‐up was similar between the 3 groups.

There were no patient deaths during the follow‐up. The incidence of VF recurrence, including electrical storm, was similar between the CAS and ERS groups (23% versus 19%; *P*=1.0). Patients with CAS and ERS exhibited similar rates of arrhythmic events (log‐rank, *P*=0.71) (Figure 7A). There was no statistically significant difference in the incidence of VF recurrence between patients with symptomatic and silent CAS (log‐rank, *P*=0.13). Antianginal drugs, such as nitrates and calcium antagonists, were used in all patients diagnosed as having CAS. Three symptomatic patients with CAS experienced VF recurrence. Two of those patients experienced VF recurrence after discontinuing treatment with antianginal drugs (before vasospasm induction test [1 patient] and self‐interruption [1 patient]). The remaining 10 patients with CAS, including all of the patients with silent CAS, received appropriate treatment without discontinuation of antianginal drugs and showed a favorable outcome (Figure 7B).

Patients in the ERS group did not receive antianginal drugs. The incidence of anterior J wave was reported in all 4 patients with ERS with VF recurrence. The use of monotherapy or combination therapy consisting of quinidine, cilostazol, and bepridil in these 4 patients with ERS was effective in suppressing VF.

## Discussion

### Main Findings

Both ERS and CAS may induce VF in patients without structural heart disease. Differentiating CAS from ERS as a cause of VF may be challenging because patients with CAS are often associated with inferolateral J wave, mimicking ERS. This was the first study evaluating the incidence of CAS, in particular silent CAS, in patients with possible ERS. Unless a spasm provocation test was performed, these patients could have been misdiagnosed as having ERS.

These results suggest that a considerable number of patients previously diagnosed as having ERS may have been cases of silent CAS. J‐ST–segment augmentation before the onset of VF, which is considered a typical electrocardiographic characteristic in ERS, was also observed in patients with CAS. Treatment with antianginal drugs is essential to prevent the recurrence of VF in patients with CAS, whereas use of quinidine, cilostazol, and bepridil was effective in patients with pure ERS. The spasm provocation test and close clinical follow‐up are considered essential to distinguish patients with silent CAS from those with ERS.

### Limited Diagnosis of CAS in Patients With Idiopathic VF and Inferolateral J Wave

In 2008, Haïssaguerre et al associated, for the first time, the J‐wave elevation in the inferolateral leads with idiopathic VF.^1^ Since then, studies have shown that the presence of a J wave is indicative of a high risk for fatal ventricular arrhythmias in patients with Brugada syndrome^17^ and acute myocardial infarction.^18^

The prevalence of CAS in patients resuscitated from cardiopulmonary arrest is 7.4% in Japan^3^ and 8.6% in Western countries.^6^ In addition, inferolateral J wave has been reported in 21% to 36% of patients with CAS.^9^, ^10^ Oh et al and Inamura et al independently showed that inferolateral J wave was associated with fatal ventricular arrhythmias in patients with CAS.^9^, ^10^ Thus far, the differentiation between patients with CAS and ERS was mainly based on the absence of chest symptoms and normal CAG. In a multicenter study conducted by Haïssaguerre et al, provocation using ergonovine was performed in only 19 of 122 patients with ERS (16%).^12^ However, Kobayashi et al reported that only 3 of 12 patients with CAS with cardiac arrest experienced prodromal chest symptoms.^3^ This indicates that a considerable number of patients with CAS without chest symptoms may have been included in cases with idiopathic VF and inferolateral J wave.

This study revealed that 38% of the patients with idiopathic VF and inferolateral J wave (ie, possible ERS) were actually patients with CAS who would not have been recognized unless spasm provocation tests were performed. In addition, ≈40% of the patients with CAS with prior VF and inferolateral J wave did not experience chest symptoms before VF. Among the 26 patients without prodromal chest symptoms, 5 (19%) were shown to have CAS. This finding suggests that a considerable number of patients with silent CAS may have been included in ERS cases, in whom a spasm provocation test was not routinely performed.

### Similarities Between CAS and ERS in Electrocardiographic Features Before VF

J‐wave augmentation before the onset of VF is considered a typical electrocardiographic feature in patients with ERS.^1^, ^12^ However, in addition to J‐wave augmentation, QRS interval widening and J‐ST–segment elevation were also documented in previously reported cases of ERS, in accordance with electrocardiographic characteristics observed in acute ischemia settings.^9^, ^10^, ^19^ In this study, available ECGs before the onset of VF in 3 patients with CAS also showed specific J‐wave and QRST characteristics, in agreement with previous ERS studies.^1^, ^12^ Inamura et al showed that the J wave was augmented in the leads associated with ischemic areas, caused by acetylcholine‐induced vasospasm.^10^ This suggests that previously reported cases of ERS may have included those with silent CAS and the J‐wave augmentation may be related to ischemic events. On the other hand, the morphological features of the J‐ST segment previously remained unchanged in most patients with ERS immediately before the onset of VF.^20^

### VF Recurrence and Treatment of ERS and CAS

Treatment with calcium antagonists and/or nitrates leads to a good prognosis for CAS.^13^ However, patients with CAS with aborted sudden cardiac arrest have been linked to a poor prognosis compared with those without such a condition.^8^, ^21^ Ahn et al demonstrated that patients with CAS with a history of aborted sudden cardiac death remain at high risk despite antianginal therapy.^21^ They reported that the annual incidence rate of recurrent ventricular tachyarrhythmia was ≈3%.^21^ Takagi et al showed that the incidence of cardiac death was significantly increased in patients for whom medications were reduced or discontinued.^8^ As shown in Figure 7A, the incidence of VF recurrence in the CAS group is consistent with that reported in previous studies.^8^, ^21^ In the current study (Figure 7B), 3 symptomatic patients with CAS experienced VF recurrence, with 2 of those experiencing VF recurrence after discontinuation of antianginal therapy. In contrast, patients with CAS receiving appropriate treatment with antianginal medications (including all of the patients with silent CAS) showed a favorable outcome. Implantation of an ICD in patients with CAS and prior VF remains controversial. Komatsu et al demonstrated that, among out‐of‐hospital cardiac arrest survivors, patients with CAS without provocable ventricular arrhythmias may be at low risk for VF recurrence.^22^ Thus, programmed ventricular stimulation may contribute to risk stratification for the recurrence of VF in patients with CAS. Further studies are warranted to confirm the effect of ICD therapy in improving the prognosis of patients with CAS with prior VF.

Quinidine and isoproterenol are suggested as first‐line therapy for suppressing VF in patients with ERS.^1^ Previous evidence has shown that patients with ERS with an anterior J wave are associated with high VF recurrence rates, and these patients responded to quinidine and isoproterenol treatment.^15^, ^23^ Moreover, bepridil and cilostazol were also effective against VF recurrence in patients with ERS.^15^, ^23^ The clinical management of CAS and ERS is different; therefore, it is essential to distinguish patients with CAS from those with ERS. A spasm provocation test and close follow‐up are necessary in patients in whom ERS is suspected.

### Study Limitations

This was a single‐center study using a retrospective analysis. First, the small number of patients may limit the interpretation of these results. Nevertheless, the number of patients with ERS and CAS with documented VF is comparable to that reported in previous multicenter studies. Second, it was not possible to obtain ECGs before and during VF in patients with silent CAS. However, their ECGs are expected to have shown similar characteristics (ie, a dynamic J‐wave augmentation, QRS widening, or J‐ST elevation) to those of patients with symptomatic CAS. Third, although spasm provocation test has been reported to be highly sensitive and specific,^24^ the possibility of false‐positive results must be acknowledged. Fourth, the spasm provocation test was not performed in 5 patients. These were considered patients with CAS because of the spontaneous ST changes associated with chest pain observed at the first VF. We were concerned about the induction of an electrical storm attributable to severe CAS by spasm provocation test. Last, CAS is more common in Japan than in Western countries. Therefore, these results may not be applicable globally. Prospective multicenter studies including more patients with ERS and CAS are warranted to confirm these findings.

## Conclusions

Approximately 40% of the patients with CAS with prior VF and inferolateral J wave did not experience chest symptoms at the first VF, and could be misdiagnosed as having ERS. This accounted for 19% of possible patients with ERS without prodromal chest symptoms at the time of VF. The management of CAS and ERS is different; therefore, it is important to differentiate the 2 conditions for optimal medical management.

## Sources of Funding

This work was supported by the Japan Society for the Promotion of Science through a Grant‐in‐Aid for Scientific Research (KAKENHI Grant JP17K09545) and the Suzuken Memorial Foundation.

## Disclosures

None.
